# Success rate of proximal tooth-coloured direct restorations in primary teeth at 24 months: a meta-analysis

**DOI:** 10.1038/s41598-020-63497-4

**Published:** 2020-04-14

**Authors:** Antonio J. Ortiz-Ruiz, Nuria Pérez-Guzmán, María Rubio-Aparicio, Julio Sánchez-Meca

**Affiliations:** 1 Department of Integral Paediatric Dentistry. Faculty of Medicine, University of Murcia, Murcia, Spain; 2 Department of Health Psychology, Faculty of Health Sciences, University of Alicante, Alicante, Spain; 3 Department of Basic Psychology & Methodology, Faculty of Psychology, University of Murcia, Murcia, Spain

**Keywords:** Glasses, Paediatric research, Biomaterials

## Abstract

The aim was to determine the survival of tooth-coloured restorative materials in proximal restorations of primary teeth at 24 months of follow-up and the influence of the following variables: use of coating, use of cavity conditioner, use of rubber dam isolation, the cavity form, the dentist’s experience and the methodological characteristics of the studies. We conducted a search until May 2019, obtaining 16 articles from which 30 independent studies were extracted, which were considered as units of analysis. Four outcome measures were extracted from each study: retention, marginal integrity, anatomic form, and absence of recurrent caries. Separate meta-analyses were carried for each outcome and multiple meta-regression model was applied. The outcomes with the highest mean success rates were absence of recurrent caries and anatomic form. The type of material significantly influenced success rates. The best materials were resin-based material plus total-etching adhesion and resin-modified glass ionomer cement (RMGIC), and the worst high viscosity glass ionomer cement (HVGIC). Atraumatic restorative treatment (ART) had a lower success rate than the conventional cavity form. RMGIC had the best clinical performance and HVGIC the worst. The form of the cavity, blinding and the experience of the operator were the variables that influenced success rates. Proximal primary molar restorations should be performed with RMGIC as it combines good mechanical performance of the resins together with the prevention of secondary caries of glass ionomers.

## Introduction

The Global Burden of Disease 2015 study^1^ concluded that oral health has not improved in the last 25 years: the age-standardized prevalence rate of untreated caries in primary teeth was 7.8% (573 million children) and 126 million children worldwide had incident cases of caries in primary teeth in 2015. Dental caries, according to the WHO, remains the most frequent chronic disease in early childhood in most communities around the world, having a negative impact on the quality of life of both the child and their family, and is considered a public health problem^2,3^.

The toxic effects on the patient, health professionals and the environment, the increasing prevalence of minimal intervention restorative approaches and the increased demand for aesthetics, have reduced the use of amalgam as the material of choice for the restoration of primary teeth^4,5^. Currently, restorative treatments for primary teeth include a wide variety of tooth-coloured materials such as glass ionomer cements (GIC), resin-modified glass ionomer cement (RMGIC), high viscosity glass ionomer cement (HVGIC), compomers and composite resins (CR). In general, composites are recommended in children with a low risk of caries, compomers in children with a moderate risk and glass ionomers in children with a high risk^6^.

GICs are good materials for the primary dentition^7^ due to their ability to adhere to the dental structure, low polymerization contraction, lack of postoperative sensitivity, biological compatibility and the anti-cariogenic effects of fluoride release^8^. However, their low resistance to fracture and wear does not make them suitable materials for proximal restorations, with a failure rate of 6.6–60% at 36 months^9^. RMGICs, whose composition includes resin, improve the physical and aesthetic properties of GICs, while maintaining their potential for fluoride release^10–12^. HVGICs maintain the mechanical properties offered by resins (microhardness, resistance to abrasion and fracture) without containing resins, and have performed well in reconstructions of the posterior sector in primary and permanent dentition^13,14^.

CRs have been used as a substitute for amalgams, with good short-term performance in both occlusal and proximal restorations. However, due to the problems derived from polymerization contraction (loss of retention, microfiltration and secondary caries) and because these materials are very sensitive to the technique used and require a demanding placement protocol^15^, this has led paediatric dentists to search for alternatives. Compomers and giomers, which present the mechanical and aesthetic properties of a composite together with the ability to release fluoride, have been used in proximal cavities of primary teeth^16–20^.

Paediatric dentists have, therefore, a wide range of materials for the restoration of proximal cavities, or class II, in primary teeth. In choosing the material, one important factor should be considered: the longevity/survival of the restoration, since the replacement of failed restorations is a problem for patients, professionals and public health systems^19^. The success rate of the restoration depends on the properties of the material, the level of the risk of caries, the state of the tooth affected, the characteristics of the patient and the dentist’s ability in the use of the materials and in handling the child’s behaviour^15,21^.

A 2007 meta-analysis of 21 studies concluded that, of the coloured restorations in primary molar proximal lesions followed for at least 1 year, RMGIC had the highest clinical success rates, although only one RMGIC product was assessable^22^. A 2009 systematic review studied all types of restorative materials used in primary dentition [silver amalgam, GICs, silver reinforced glass ionomer cements (SRGICs), RMGICs, CR, stainless steel crowns and compomers], all types of cavities (classes I to V) and different cavity preparations [decay removed using drills, and ART removing decay with manual instruments only] with a follow up of >6 months. They could only include three studies and these did not provide sufficient evidence to make any recommendations about which filling material to use^21^. A systematic review and meta-analysis, published in 2016, analysed the survival time of adhesive restorations (CR, GIC, RMGIC, SRGIC, and compomer) for class I and II of primary molars, and concluded that there was weak evidence that adhesive materials with a resin component have similar survival rates for 24 months and up to 48 months and that there was no evidence that adhesive materials with a resin component in the formula had a greater survival rate than glass ionomer cement^24^. A systematic review studied, in 2018, the survival of restorations (class I, class II, and crown) placed using different materials in primary teeth with at least one year of follow-up. They found large variations in the annual failure rates (0–29.9%) due to the differences in the techniques and material evaluated. The lowest annual failure rate was in class I restorations using a rubber dam and those using CR (1.7–12.9%) and the highest success rate was for stainless steel crowns (96.1%)^21^.

It is necessary to determine the success rate of tooth-coloured materials used in the restorations of proximal classes of temporary teeth, including those marketed in recent years, such as HVGIC and giomers. Therefore, the main objective of this meta-analysis was to determine which tooth-coloured restoration material is most suitable for proximal fillings in primary teeth. The question posed was: What will the survival of different tooth-coloured restorative materials in proximal restorations of primary teeth be at 24 months? The secondary objective was to determine factors influencing the success of the tooth-coloured restoration material at 24 months. The null hypothesis of our study was that the success rate at 24 months of proximal restorations of primary teeth does not depend on the tooth-coloured material used.

## Materials and methods

The meta-analysis was carried out according to the PRISMA (Preferred Reporting Items for Systematic Reviews and Meta-analyses) method^25^. Supplementary File 1 shows the PRISMA checklist. The protocol was registered in the PROSPERO international database for systematic reviews (CRD42019138646).

### Inclusion and exclusion criteria

Using the components of the Participants, Interventions, Comparisons, Outcomes, and Study designs (PICOS) system^26^, the studies to be included in this meta-analysis the studies had to meet the following criteria:

- Participants: primary teeth with proximal caries in children aged 2–14 years.
- Interventions: tooth-coloured proximal (or class II) restorations (composites, compomers, RMGIC, HVGIC and giomers) evaluated *in vivo*.
- Comparisons: Not applicable.
- Outcomes: Success rate at 24 months of follow-up for retention, marginal integrity, anatomic form, and absence of recurrent caries.
- Study designs: randomized controlled trials (RCTs), non-randomized controlled trials (nRCTs), and uncontrolled trials.

In addition, published and unpublished studies were accepted. We excluded reviews, clinical cases, *in vitro* studies, observational studies, studies of permanent teeth, studies evaluating the survival of materials in classes I, III, IV and V, studies dealing only with amalgam or cermet restorations or stainless steel crowns, and studies with a follow up other than 24 months.

### Search strategy

We exhaustively searched the following electronic databases: PubMed, MEDLINE, SciELO, Embase, Scopus, WOS, LILACS and BBO. We also carried out a manual search to find studies not included in the electronic databases. We adapted the search strategy to the requirements of each database. The references of the studies recovered were also reviewed to identify studies that might fulfil the selection criteria. The search languages were English, Spanish and Portuguese, and the search covered 1985 to May 2019. We included the following search terms, making the appropriate adaptations to the language required by the different databases: “survival”, “durability”, “primary”, “deciduous”, “teeth”, “class II”, “proximal”, “occlusoproximal”, “composite”, “compomer”, “glass ionomer”, “high viscosity glass ionomer”, “resin modified glass ionomer” and “giomer”.

Studies were selected in a three-stage procedure. First, the title of the study was considered. Studies that appeared in the results of more than one database were only taken into account once, and all duplicate studies were eliminated. Secondly, we read the abstracts of the articles. If the summary did not provide sufficient information to make a decision about its inclusion or exclusion, we reviewed the entire study before making the final decision. Thirdly, we considered the full text to determine its inclusion or exclusion. Selection was made independently by two authors (AJOR, NPG). Subsequently, the authors discussed studies in which there were discrepancies until a consensus was reached.

### Data extraction

The most relevant data were extracted from the articles and included in a database which collected the main characteristics of the intervention and the evaluation criteria of the restoration materials. The complete database is shown in Supplementary Dataset file. For the extraction of the results, within the same article, each restoration material, or the same material under different experimental conditions, was considered as an independent study.

All materials were classified into five types: resin-based materials bonded using total etching (which includes: composites, giomers, compomers, fluid composites); resin-based materials bonded with a self-etching adhesive (composites, compomers, fluid composite); open-sandwich technique (RMGIC as base plus composite); RMGIC; and HVGIC.

To compare the results of the studies, the evaluation criteria of the different systems used were unified into four categories (Supplementary File 2). These systems were: United States Public Health Service (USPHS) criteria by Ryge and Cvar in 1971^11,27^, USPHS modified by Ryge and Snyder in 1973^28^, USPHS modified by Ryge in 1980^12,18,29–32^, USPHS modified by Van Dijken in 1986^16,33–35^, USPHS modified by Ryge in 1980 and ART modified criteria^36^, own system^35^, FDI criteria^20^.

The four evaluation categories used were:

- Retention. No detachment of the material and no partial or total fracture that required repair or a new restoration.
- Marginal integrity. No discoloration, filtration or defects in marginal adaptation.
- Anatomic form. No alterations in the shape and texture of the surface of the restoration.
- Absence of secondary caries.

The most relevant characteristics of the intervention studied were:

- Conditioning of the cavity, referring only to glass ionomer materials.
- Coating (only glass ionomers materials).
- Complete isolation with rubber dam.
- Cavity form, differentiating between cavities made according to ART principles (manual cavities made with spoon) and conventional cavities (cavities made by using rotary instruments and burrs).
- Operator experience, considering non-graduate student as inexperienced and graduate dentists as experienced.

The following patient characteristics were extracted: mean and SD of age (in years) and sex (% male). The following methodological characteristics were extracted: design (RCT, nRCT, or uncontrolled trial), random assignment, blinded assessment, reporting bias, and sample size. Financial sources and year of the study were also extracted. To check the reliability of the extraction of the study characteristics, two independent coders doubly coded all studies. The results were highly satisfactory overall, with kappa coefficients ranging between 0.927 and 1.0 (mean = 0.993) for categorical variables, and intra-class correlations between 0.805 and 1.0 (mean = 0.979) for continuous variables. Inconsistencies between the coders were resolved by consensus.

### Statistical analysis

From each sample, the proportions of success at 24 months were extracted for the four outcome measures: retention, marginal integrity, anatomic form, and absence of recurrent caries. Separate meta-analyses were made for each outcome. To normalize the distribution of the success rates, they were transformed using the logit event rate: *Lp* = *Ln* [*p*/(1 − *p*)], with *p* being the success rate, *Ln* the natural logarithm, and *Lp* the logit event rate. In each meta-analysis, a random-effects model was assumed^38^ and, consequently, the logit event rates were weighted by the inverse variance, defined as the sum of the within-study and between-studies variance. The latter was estimated using the DerSimonian and Laird method^39^. The sampling variance of each logit event rate, *V* (*Lp*), was calculated as: *V* (*Lp*) = 1/(*np*) + 1/[*n* (1 − *p*)], with *n* being the sample size. Subsequently, the results were back-transformed to success rates to facilitate interpretation by means of: *p* = *e* ^*Lp*^/(*e* ^*Lp*^ + 1), with *e* being the base of the natural logarithm^40^. In each meta-analysis, the 95% confidence limits around the mean success rate were computed using the method proposed by Hartung^41^. The heterogeneity of the success rates was assessed by constructing a forest plot and calculating the *Q* statistic and the *I*^2^ index. *I*^2^ values of about 25%, 50%, and 75% can be considered as reflecting low, moderate, and high heterogeneity^42^. To test whether publication bias was a threat to the validity of the meta-analytic results, funnel plots were constructed applying the trim-and-fill method^43^.

If studies were found to have heterogeneity, moderator analyses were carried out using meta-regressions and weighted ANOVAs for continuous and categorical variables, respectively, assuming a mixed-effects model^44,45^. The improved *F* statistic proposed by Knapp and Hartung^46^ was applied to test the significance of the moderator variables. *Q*_E_ and *Q*_W_ statistics were applied to examine model misspecification for the continuous and categorical moderators, respectively. The proportion of variance accounted for by the moderator variables was estimated using *R*^2^, an index that takes into account the total and residual between-studies variance^47^. Finally, to identify the study characteristics that best explained variability of the success rates, a multiple meta-regression model was applied. The moderator variables included in the model were selected taking into account both statistical and practical significance achieved in the previous analyses. All statistical analyses were carried out with the metafor package for R^48^.

## Results

### Results of the systematic search

**Figure 1:**
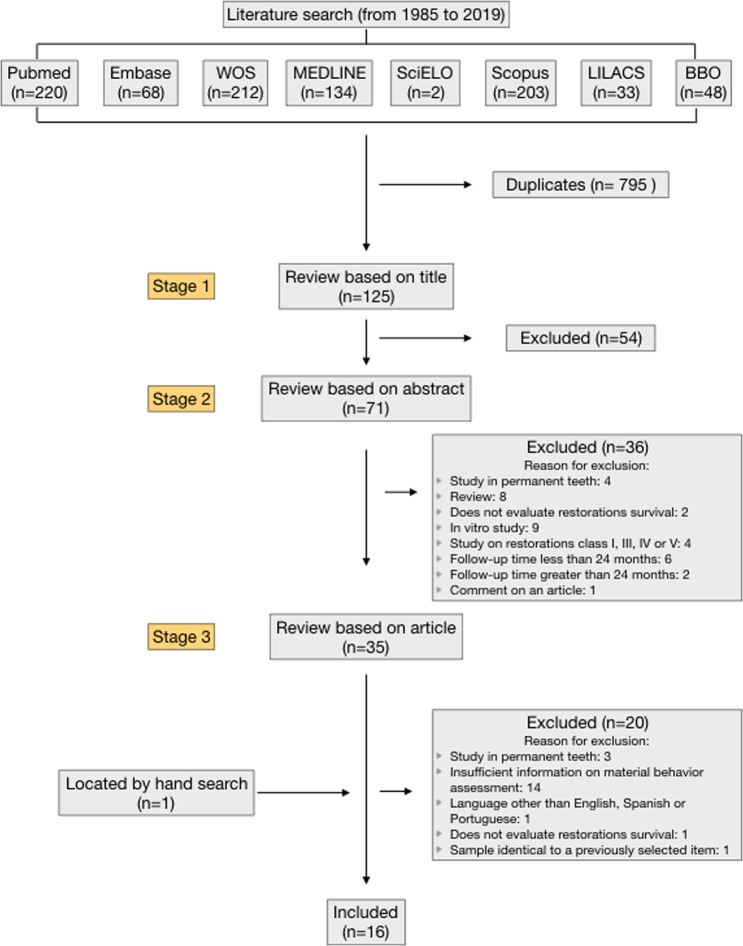
Flow chart for search strategy.

Figure 1 shows a flowchart of the selection process. The search strategy yielded 920 articles: 220 from PubMed, 68 from Embase, 212 from WOS, 134 from MEDLINE, 2 from SciELO, 203 from Scopus, 33 from LILACS and 48 from BBO. After excluding 795 duplicate articles, 125 articles remained.

Seventy-one articles were accepted due to the title and, after reading the corresponding abstracts, 36 were excluded, due to:

- Study in permanent teeth: 4.
- Systematic review: 8.
- Did not evaluate restoration survival: 2.
- *In vitro* study: 9.
- Study of restorations class I, III, IV or V: 4.
- Follow-up time <24 months: 6.
- Follow-up time >24 months: 2.
- Comment on an article: 1.

After complete reading of the remaining 35 articles, 20 were excluded due to:

- Study in permanent teeth: 3^49–51^.
- Insufficient information on evaluation of material performance: 14^10,19,52–63^.
- Language other than English, Spanish, Portuguese: 1. (Summary in English, full article in Korean)^64^.
- Did not evaluate restoration survival: 1^65^.
- Identical to a previously selected item: 1. One article was found with a different title but with the same authors and an identical sample. We excluded one of the two articles^66^.

One more article was located by manual search.

Finally, 16 articles were selected for review^11,12,16,18,20,27–37^.

### Descriptive characteristics of the studies

The 16 articles included in the meta-analysis were published over a 20-year period. The first, carried out by Andersson-Wenckert *et al*.^28^, was published in 1995, and the last, by Sengul and Gurbuz^20^, in 2015. Seven were randomized controlled studies^11,18,27,32,34,36,37^, five were controlled non-randomized trials^12,20,29–31^, and four were uncontrolled, non-randomized trials^16,28,33,35^. Randomization was correct in seven^11,18,27,32,34,36,37^ articles and incorrect or undefined in the remaining nine^12,16,20,28–31,33,35^. Among the articles with correct randomization, this was done by computer in three^18,27,32^.

In nine studies, the dentists who evaluated the restorations were unaware of the material used, as they were triple blinded^11,12,16,31,32,34,36,37^. In 14 articles there was no reporting bias^11,12,16,18,20,27,29,31–37^, and 15 articles did not declare whether there were conflicts of interest or not^11,12,16,18,20,27–31,33–37^. The source of funding was public in six of the articles^11,16,28,33,34,37^ and private or mixed in the remaining 10^12,18,20,27,29–32,35,36^. Thirty independent studies were extracted from the 16 articles selected. The most important characteristics of each study are shown in Table 1.

**Table 1:** Main results of the studies.

Study	Participants (number of children, average age and interval)	Materials (n)	Cavity conditioner (*)	Coat	Cavity form	Dentist experience	Rubber dam	Retention success rate	Marginal integrity success rate	Anatomic form success rate	Absence of recurrent caries success rate
**Andersson-Wenckert et al**^28^.	25 children. ā: 8[6–10]	ChemFil II n = 44	Durelon liquid (40% polyacrylic acid)	yes (protecting varnish)	Conventional	Experienced	No	0.86	—	0.86	0.9
**Andersson-Wenckert** ***et al****.*^16^	79 children. ā: 8[5–12]	Dyract n = 113	Dyract primer	No	Conventional	Experienced	No	0.8	0.95	0.96	0.95
**Folkesson** ***et al****.*^33^	85 children. ā: 7.8[4–12]	Vitremer™ n = 134	Vitremer™ Primer	No	Conventional	Experienced	No	0.96	0.95	0.96	0.95
**Espelid** ***et al****.* **(1999)**^29^	43 children. ā: 7.8 ± 1.5[5–11]	Vitremer™ n = 44	No	Vitremer™ Gloss	Conventional	Experienced	Not specified	0	0	—	2.27
**Attin** ***et al****.* **(2000)a**^30^	52 children [3.8 – 10.6]	TPH Spectrum + Prime-Bond™ 2.0 n = 71	—	—	Conventional	Experienced	no	0.97	0.94	1	0.97
**Attin** ***et al****.* **(2000)b**^30^	52 children [3.8 – 10.6]	Compoglass^®^ + Single Component Adhesive n = 70	—	—	Conventional	Experienced	no	0.94	0.93	1	1
**Lo** ***et al****.* **(2001)a**^36^	[6–14]	ChemFlex™ n = 13	IV liquid diluted 50% with H_2_O	No	ART	Experienced	no	41.6	8.3	8.3	0
**Lo** ***et al****.* **(2001)b**^36^	[6–14]	Fuji IX GP n = 13	IV liquid diluted 50% with H_2_O	No	ART	Experienced	no	30.8	7.7	15.4	2.6
**Kavvadia** ***et al****.* **(2004)**^18^	75 children ā: 7 ± 1.2[6–9]	F2000 + Clicker n = 57	—	—	Conventional	Experienced	Yes	1	1	1	0.96
**Ersin** ***et al****.* **(2006)a**^12^	219 children ā: 8.07 ± 1.51[6–10]	Fuji IX GP n = 72	GC Dentin Conditioner	Fuji Varnish	ART	Experienced	No	0.75	0.75	0.75	0.72
**Ersin** ***et al****.* **(2006)b**^12^	219 children ā: 8.07 ± 1.51[6–10]	Surefil + Xeno III n = 75	—	—	ART	Experienced	No	0.81	0.81	0.81	0.75
**Anderson-Wenckert** ***et al****.* **(2006)a**^34^	57 children. ā: 8[5–11]	Vitremer™ n = 53	Vitremer™ Primer	No	Conventional	Experienced	no	3.77	4	8.2	3.77
**Anderson-Wenckert** ***et al****.* **(2006)b**^34^	57 children. ā: 8[5–11]	Tetric Flow^®^ + Excite^®^ n = 54	No	No	Conventional	Experienced	yes	3.70	6.1	4.1	8.0
**Anderson-Wenckert** ***et al****.* **(2006)c**^34^	24 children. ā: 8[5–10]	Tetric Flow^®^ + Excite^®^ n = 22	No	No	Conventional	Experienced	yes	4.5	4.3	0	0
**Anderson-Wenckert** ***et al****.* **(2006)d**^34^	57 children. ā: 8[5–11]	Tetric Flow^®^ + Prompt™ L-Pop™ n = 24	No	No	Conventional	Experienced	yes	0	0	0	0
**Atieh M. (2008)**^27^	87 children. ā: 5.5 ± 1.1[4–7]	Vitremer™ + Filtek™ Z250 (Open sandwich) n = 64	No	No	Conventional	Experienced	yes	4.68	3.1	—	3.1
**Ersin** ***et al****.* **(2008)a**^31^	126 children. ā: 7.6[6–8]	Ketac Molar n = 57	Ketac Conditioner	Ketac Molar Glaze	ART	Experienced	No	—	0.66	0.66	0.66
**Ersin** ***et al****.* **(2008)b**^31^	126 children. ā: 7.6[6–8]	Ketac Molar n = 56	Ketac Conditioner	Ketac Molar Glaze	ART	Experienced	No	—	0.62	0.62	0.60
**Topaloglu-Ak** ***et al****.* **(2009)a**^32^	327 children. ā: 6.2 ± 0.5[6,7]	Filtek™ Z250 + Adper™ Prompt™ L-Pop™ n = 210	No	No	ART	Experienced	No	68	20	—	12
**Topaloglu-Ak** ***et al****.* **(2009)b**^32^	327 children. ā: 6.2 ± 0.5[6,7]	Filtek™ Z250 + Adper™ Prompt™ L-Pop™ n = 200	Carisolv™	No	ART	Experienced	No	78	10	—	12
**Alves dos Santos** ***et al****.* **(2009)a**^11^	48 children. ā: 5.75[3–9]	Vitremer™ n = 12	Vitremer™ Primer	No	Conventional	Experienced	Yes	0	33.3	25	25
**Alves dos Santos** ***et al****.* **(2009)b**^11^	48 children. ā: 5.75[3–9]	Freedom + Stae adhesive n = 13	No	No	Conventional	Experienced	Yes	0	46.15	30.76	46.15
**Alves dos Santos** ***et al****.* **(2009)c**^11^	48 children. ā: 5.75[3–9]	TPH^®^ Spectrum^®^ + Prime & Bond^®^ NT n = 14	No	No	Conventional	Experienced	Yes	0	21.42	14.28	21.42
**Carvalho** ***et al****.* **(2010)a**^37^	232 children ā: 6.3[6,7]	GC Fuji IX n = 83	GC Fuji IX liquid diluted with a wet cotton ball	No	ART	Experienced	No	0.12	—	—	0.93
**Carvalho** ***et al****.* **(2010)b**^37^	232 children ā: 6.3[6,7]	GC Fuji IX n = 72	GC Fuji IX liquid diluted with a wet cotton ball	No	ART	Experienced	Yes	0.93	—	—	0.91
**Kotsanos** ***et al****.*^35^	61 children. ā: 6.3 ± 1.60[3,5–8]	Vitremer™ n = 83	Vitremer™ Primer	“Finishing gloss “	Conventional	Experienced	Yes	—	0.95	0.96	0.99
**Sengul** ***et al****.* **(2015)a**^20^	41 children ā: 5.8 ± 0.9[5–7]	Beautiful n = 38	—	—	Conventional	Experienced	Yes	0.9	0.94	0.92	0.92
**Sengul** ***et al****.* **(2015)b**^20^	41 children ā: 5.8 ± 0.9[5–7]	GC Fuji II LC n = 32	GC Cavity Conditioner	No	Conventional	Experienced	Yes	0.9	0.9	0.94	1
**Sengul** ***et al****.* **(2015)c**^20^	41 children ā: 5.8 ± 0.9[5–7]	Valux Plus + Prime-Bond NT n = 40	—	—	Conventional	Experienced	Yes	0.93	0.95	0.93	0.98
**Sengul** ***et al****.* **(2015)d**^20^	41 children ā: 5.8 ± 0.9[5–7]	Dyract AP n = 36	—	—	Conventional	Experienced	Yes	0.78	0.81	0.81	0.89

(*) Cavity conditioner, referring only to glass ionomer materials.

### Mean success rates and heterogeneity

**Figure 2:**
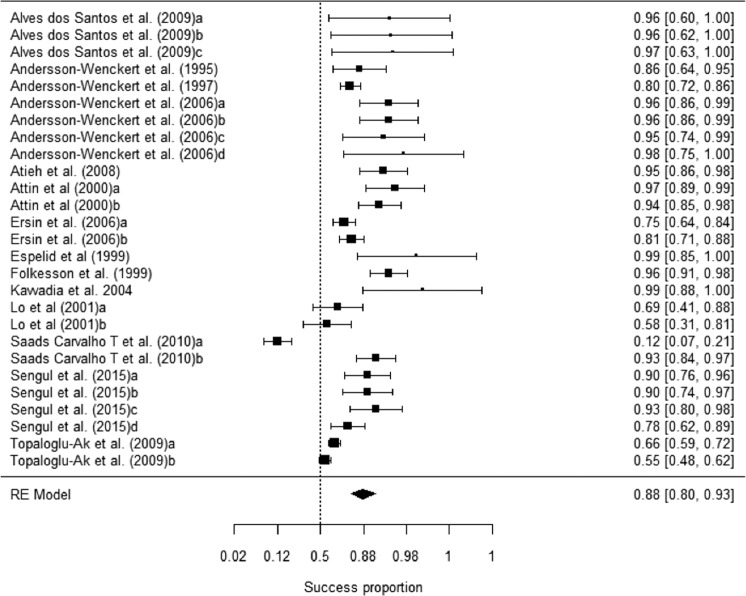
Forest plot of success rates at 24 months for retention (and 95% confidence intervals).

Table 2 shows the mean success rates, the 95% confidence intervals, and the heterogeneity statistics (*Q* and *I*^2^) for each outcome. The absence of recurrent caries and anatomic form were the outcomes with the highest mean success rates (*p*_+_ = 0.909 and *p*_+_ = 0.901, respectively). The mean success rates for marginal integrity and retention were 0.898 and 0.879, respectively. As retention was the main outcome, Fig. 2 shows a forest plot of the 27 retention success rates, and Supplementary File 3 shows the forest plots of the success rates for marginal integrity, anatomic form and absence of recurrent caries. The *Q* statistic was significant (*p* < 0.0001) for all four outcomes and the *I*^2^ indices were >75% in all cases (Table 2). The wide heterogeneity of the success rates was investigated by analysing the influence of moderator variables.

**Table 2:** Mean success rates, 95% confidence intervals, and heterogeneity statistics.

95% CI						
	*k*	*p*_+_	LL	UL	*Q*	*I*^2^
Retention	27	0.879	0.803	0.928	260.4977***	90.02
Marginal integrity	27	0.898	0.852	0.931	129.7594***	79.96
Anatomic form	24	0.901	0.845	0.938	103.8451***	77.85
Absence of recurrent caries	30	0.909	0.865	0.939	151.9184***	80.91

*k* = number of studies. *p*+ = mean success rate. LL and UL: lower and upper limits of the 95% confidence interval for *p*+. *Q* = Cochran’s heterogeneity Qstatistic; *Q* statistic has *k* – 1 degrees of freedom. I2 = heterogeneity index. ***p < 0.0001.

### Analysis of publication bias

**Figure 3:**
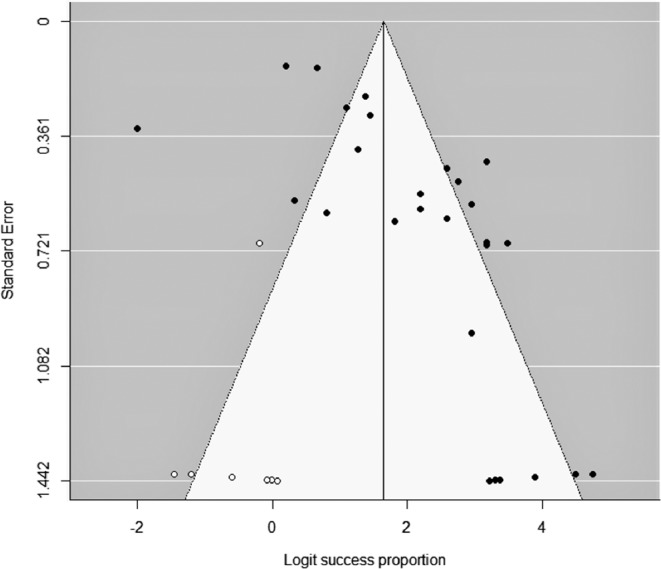
Funnel plot of the retention success-rate logits at 24 months. The seven white circles represent logits imputed using Duval and Tweedie’s trim-and-fill method.

To determine whether publication bias was a threat to the conclusions of the meta-analysis, funnel plots were constructed applying Duval and Tweedie’s trim-and-fill method. Figure 3 shows the funnel plot of the retention success rates, with a slightly higher concentration of data on the right side of the mean success rate. By applying the trim-and-fill method, seven additional success rate estimates were imputed to achieve symmetry in the funnel plot. Adding the seven success rates led to a slight decrease in the mean success rate from the original 0.879 to 0.839 (95% CI: 0.764–0.893), implying a 4.8% decrease, which may be considered negligible. Therefore, publication bias did not threaten the overall success rate for retention outcome.

Similar analyses were carried out for the other three outcomes. Supplementary File 3 presents the funnel plots for marginal integrity, anatomic form, and absence of recurrent caries. For both marginal integrity and anatomic form, five new success rates were imputed to adjust the funnel plots to symmetry (see Supplementary Figs 4 and 5, respectively). The mean success rates for marginal integrity obtained with the 27 original success rates and after imputing data, were 0.898 and 0.881 (95% CI: 0.832–0.916), respectively, with a negligible decrease of 1.9% when data imputation was applied. For anatomic form, the mean success rates for the original and once imputed data were 0.901 and 0.876 (95% CI: 0.817–0.919), respectively, implying a negligible decrease of 2.9%. With respect to the absence of recurrent caries, the trim-and-fill method added eight success rates on the left side of the funnel plot to achieve symmetry (see Supplementary Fig. 6 in File 3), leading to a decrease in the mean success rate from 0.909 (with the 24 original success rates) to 0.878 (95% CI: 0.828–0.915) once data were imputed. In this case, the decrease of 3.5% can also be considered negligible. Therefore, these results enabled publication bias to be ruled out as a threat to the validity of the meta-analytic results.

### Analysis of moderator variables

The analysis of moderator variables was carried out separately for the four outcomes. We only present here the results of the retention outcome, since the pattern of results was very similar for the remaining outcomes. However, the results of applying ANOVAs and simple meta-regressions for the marginal integrity, anatomic form and absence of recurrent caries are shown in Supplementary File 4. Table 3 presents the simple meta-regressions applied for each continuous moderator variable on the estimated retention success rates. Of the moderators analysed, only sample size was significantly negatively associated with the success rates (*p* = 0.001), accounting for 37% of variance. A marginally significant result was found for anatomic form (*p* = 0.052, accounting for 17% of variance; see Table 3 in Supplementary File 4).

**Table 3:** Results of the simple meta-regressions of continuous moderator variables on the retention success rate estimates.

Moderator variable	*k*	*b*_j_	*F*	*p*	*Q*_E_	*R*^2^
Mean age (years)	23	0.229	0.553	0.465	204.315***	0.03
SD of age (years)	11	1.193	1.848	0.207	46.612***	0.27
Sample size	27	−0.009	13.776	0.001	151.956***	0.37
Gender (% male)	16	−0.091	2.984	0.152	156.351***	0.29
Year of the study	27	−0.035	0.499	0.486	235.331***	0.03

*k* = number of studies. *bj* = regression coefficient of each predictor. *F* = Knapp-Hartung’s statistic for testing the significance of the predictor (the degrees of freedom for this statistic are 1 for the numerator and *k* – 2 for the denominator). *p* = probability level for the *F* statistic. *QE* = statistic for testing the model misspecification. *R*^2^ = proportion of variance accounted for by the predictor. ***p < 0.0001.

Regarding categorical moderators, Table 4 shows the results of the weighted ANOVAs applied to the estimated retention success rates. The type of material significantly influenced the success rates (*p* = 0.011), explaining 36% of variance. Specifically, better results were found for resin-based material plus total-etching adhesion (*p*_+_ = 0.949), RMGIC (*p*_+_ = 0.956), and open sandwich restoration (*p*_+_ = 0.950), in comparison with resin-based material plus self-etching adhesion (*p*_+_ = 0.824) and HVGIC (*p*_+_ = 0.671). A marginally significant result was also found for anatomic form (*p* = 0.051), accounting for 51% of variance (Table 4 in Supplementary File 4), with the lowest success rate shown by HVGIC (*p*_+_ = 0.759).

**Table 4:** Results of the weighted ANOVAs of qualitative moderator variables on the retention success rates.

			95% CI		
Moderator variable	*k*	*p*_+_	LL	LU	ANOVA results
-Material:					*F*(4,22) = 4.22, *p* = 0.011 *R*^2^ = 0.36 *Q*_W_(22)=148.92, *p* < 0.001
Resin-based material plus total-etching adhesion	7	0.949	0.868	0.981	
Resin-based material plus self-etching adhesion	8	0.824	0.672	0.914	
Resin-modified Glass-ionomer cement (RMGIC)	5	0.956	0.865	0.986	
High-viscosity Glass-ionomer Cement (HVGIC)	6	0.671	0.447	0.838	
Open Sandwich Restoration	1	0.950	0.639	0.995	
-Use of coat:					*F*(1,10) = 0.16, *p* = 0.699 *R*^2^ = 0.0 *Q*_W_(10)=149.51, *p* < 0.001
No	9	0.854	0.609	0.956	
Yes	3	0.904	0.474	0.989	
-Use of cavity conditioner:					*F*(1,10) = 2.27, *p* = 0.163 *R*^2^ = 0.03 *Q*_W_(10)=135.14, *p* < 0.001
No	2	0.973	0.695	0.998	
Yes	10	0.826	0.605	0.937	
-Use of rubber dam isolation:					*F*(1,22) = 2.25, *p* = 0.148 *R*^2^ = 0.16 *Q*_W_(22)=206.44, *p* < 0.001
No	14	0.847	0.726	0.920	
Si	10	0.931	0.834	0.973	
-Cavity form:					*F*(1,25) = 24.76, *p* < 0.001 *R*^2^ = 0.46 *Q*_W_(25)=138.88, *p* < 0.001
Atraumatic restorative treatment (ART)	8	0.649	0.488	0.783	
Conventional cavity design	19	0.936	0.895	0.961	
- Dentist experience:					*F*(1,25) = 4.34, *p* = 0.048 *R*^2^ = 0.07 *Q*_W_(25)=232.264, *p* < 0.001
Experienced	25	0.895	0.828	0.938	
Non-experienced	2	0.554	0.168	0.885	
**−Type of study:**					
Noncontrolled trial	3	0.892	0.614	0.977	*F*(2,24) = 0.25, *p* = 0.783 *R*^2^ = 0.09 *Q*_W_(24)=197.82, *p* < 0.001
Nonrandomized controlled trial	9	0.899	0.768	0.961	
Randomized controlled trial	15	0.854	0.718	0.931	
-Random assignment:					*F*(1,25) = 0.55, *p* = 0.465 *R*^2^ = 0.16 *Q*_W_(25)=197.88, *p* < 0.001
No/Incorrect	12	0.897	0.792	0.952	
Correct	15	0.851	0.716	0.929	
-Triple-blind					*F*(1,25) = 5.48, *p* = 0.028 *R*^2^ = 0.32 *Q*_W_(25)=176.25, *p* < 0.001
No	11	0.934	0.859	0.971	
Yes	16	0.805	0.674	0.892	
-Reporting bias:					*F*(1,25) = 0.82, *p* = 0.374 *R*^2^ = 0.08 *Q*_W_(25)=236.27, *p* < 0.001
No	24	0.867	0.779	0.924	
Yes	3	0.935	0.726	0.987	
-Financial source:					*F*(1,15) = 1.07, *p* = 0.317 *R*^2^ = 0.0 *Q*_W_(15)=157.82, *p* < 0.001
Private or mixed	5	0.807	0.482	0.949	
Public	12	0.909	0.785	0.965	

*k* = number of studies. *p*+ = mean success rate. LL and LU = lower and upper 95% confidence limits for *p*_+_. *F* = Knapp-Hartung’s statistic for testing the significance of the moderator variable. *Q*W = statistic for testing the model misspecification. *R*^2^ = proportion of variance accounted for by the moderator.

The form of the cavity was also associated with the success rates (*p* < 0.001), accounting for 46% of variance: the mean retention success rate was lower for ART (*p*_+_ = 0.649) than for conventional cavity design (*p*_+_ = 0.936). Similar results were found for marginal integrity (*p* = 0.040, *R* ^2^ = 0.21; Table 2 in Supplementary File 4), anatomic form (*p* = 0.001, *R* ^2^ = 0.63; Table 4 in Supplementary File 4), and absence of recurrent caries (*p* = 0.046, *R* ^2^ = 0.16; Supplementary Table 6 in File 4). The dentist’s experience was significantly associated with retention success rates (*p* = 0.048; Table 4), although it only explained 7% of variance. More experienced dentists had better success rates than non-experienced ones (*p*_+_ = 0.895 vs.0.554, respectively). Of the methodological variables analysed, assessor blinding was significantly associated with retention success rates (*p* = 0.028, *R*^2^ = 0.32; Table 4), with lower retention success rates when the assessor was blinded. These results were repeated for marginal integrity (*p* = 0.023, *R*^2^ = 21; Table 2 in Supplementary File 4), anatomic form (*p* = 0.032, *R*^2^ = 0.33; Table 4 in Supplementary File 4), and absence of recurrent caries (*p* = 0.002, *R*^2^ = 0.28; Supplementary Table 6 in File 4).

### Explanatory models

Although some of the moderators were significantly associated with retention success rates, none showed non-significant results in the model misspecification tests (*Q*_E_ and *Q*_W_ for meta-regressions and ANOVAs, respectively), suggesting residual heterogeneity among the success rates after including the moderator. Similar results were found for marginal integrity, anatomic form, and recurrent caries success rates (see Supplementary File 4). Therefore, multiple meta-regression models including the most relevant characteristics of the studies were applied to explain the variability in the different success rates.

The predictors included in the meta-regression models for explaining success rates were selected as a function of both statistical and practical significance achieved in the previous results of the ANOVAs and simple meta-regressions. A moderator variable was included in the model when the *F* statistic was significant and the *R*^2^ index was >30%. With respect to retention success rates, four predictors were included: the sample size, the material (dichotomized as: 1 for resin-based material plus total-etching adhesion, RMGIC, and open sandwich restoration; and 0 for resin-based material plus self-etching adhesion and HVGIC), the form of the cavity (0: ART, and 1: conventional cavity design), and assessor blinding (0: no blinding, and 1: blinding).

Due to missing data in some variables, the number of studies included in the meta-regression was *k* = 27. The results are shown in Table 5. The full model showed a significant association with the retention success rates (*p* = 0.001), accounting for 45% of variance. However, once the other predictors were controlled for, none showed a significant association with the success rates. This may be due to collinearity among the predictors. Inspection of the bivariate correlations between the predictors showed a significant association between the form of the cavity and the type of material (*r* = 0.66, *p* < 0.001), as ART usually uses HVGIC material. When the form of the cavity was removed from the meta-regression model, the type of material (dichotomized) was significant (*p* = 0.029) as was the full model, *F* (3, 23) = 7.34, *p* = 0.001, accounting for 49% of the variance. Sample size and assessor blinding were not significant in the multiple meta-regression model.

**Table 5:** Results of the multiple meta-regression model applied on the retention success rates, taking as predictors the sample size, the type of material (dichotomized), the cavity form, and the assessor blinding (*k* = 27).

Predictor variable	*b*_j_	*t*	*p*	Model fit
Intercept	−1.942	−0.89	0.384	*F*(4, 22) = 6.62, *p* = 0.001
Sample size	−0.0002	−0.06	0.956	*R*^2^ = 0.45
Type of material	0.831	1.51	0.146	*Q*_E_(22) = 114.86, *p* < 0.0001
Cavity form	1.558	1.68	0.108	
Assessor blinding	−0.11	0.19	0.845	

*b*_j_ = regression coefficient of each predictor. *t* = statistic for testing the significance of the predictor (with 22 degrees of freedom). *p* = probability level for the *t* statistic. *F* = Knapp-Hartung’s statistic for testing the significance of the full model. *Q*_E_ = statistic for testing the model misspecification.

The results of the multiple meta-regressions for marginal integrity, anatomic form, and recurrent caries success rates are shown in Supplementary Tables 7–9 in File 4. With respect to marginal integrity, two moderators were included in the model: cavity form and assessor blinding. There was a trend to significance for the full model (*p* = 0.051), with 22% of variance accounted for, while neither predictors were significant once the influence of each on the other was controlled for. Cavity form and assessor blinding were included in the meta-regression model for anatomic form success rates. A significant association was found for the full model (*p* = 0.005) and 61% of variance was accounted for. In addition, the cavity form was significantly associated with anatomic form success rates (*p* = 0.017) after controlling for assessor blinding. With respect to recurrent caries, three moderators were included in the meta-regression model: use of coat, cavity form, and assessor blinding. The full model was significant (*p* = 0.007), with 83% of variance accounted for. Of the three moderators, only the use of coat was negatively associated with recurrent caries success rates (*p* = 0.022).

## Discussion

Since the FDA advised, in July 2010, that dental amalgam should not be used in children aged <6 years, due to its greater sensitivity to the potential toxic effects of mercury^67^, and the EU banned its use, from July 1, 2018, for the restoration of primary teeth, in children aged <15 years, and pregnant or breastfeeding women^68^, paediatric dentists need to know what the best alternative material for the restoration of primary teeth is. Therefore, this meta-analysis tried to answer the question: which tooth-coloured restoration material has the best clinical behaviour in proximal restorations of primary teeth at 24 months? We chose proximal restorations because they have the highest failure rate, especially when functional, due to the presence of antagonistic teeth^52^.

We studied the success rate at 24 months ought to the dropout rates of study subjects increase over time because there is a marked increase in the rates of physiological exfoliation, characteristic of childhood growth^53^. Moreover, after that time many studies have reported a high level of failure, depending on clinical variables and patient related factors. In general, the annual failure rate was 17% for restorations in primary molars^69^, although some studies recorded losses of around 50% at 24 months of follow up^23,57^. The success rate for class II in primary teeth was 68% at 18 months^70^ and 52´58% at 36 months^71^. This way, a study found that after a 7-year follow-up, only 1% of initial restorations completed the study^72^.

For better understanding of the results, the materials used in the studies analysed were divided into five groups: materials containing resin bonded with total etching (composite, giomers, compomer and fluid composite); resin-containing materials bonded with a self-etching adhesive (composite, compomer and fluid composite), RMGLC, HVGIC, and open sandwich technique (RMGIC as a base and composite as a restorative material). Regardless of the material used, retention of proximal restorations was the most affected, with 12.5% of restorations lost within 24 months of placement. Marginal integrity, conservation of the anatomical shape and the absence of secondary caries, in descending order, were affected to a lesser extent.

The success of a restoration depends on factors such as the material used, the state of the tooth, the experience of the operator and, the patient’s collaboration. This last aspect is of paramount importance in paediatric dentistry, since children’s behaviour largely determines the selection of the material and the technique to be used to restore a tooth, conditioning, finally, the wide variations in the success rate between the different materials and studies^21^. Unifying all the materials included in the study in materials that contain resin and those that do not, the meta-analysis showed that those containing resin had a higher success rate in the four clinical categories studied. RMGIC had the highest success rate followed by resin-based materials used with total etching and self-etch adhesives. The material with the lowest success rate was HVGIC. Although only retention was significant, the trend in all clinical categories studied (marginal integrity, anatomic form and recurrent caries) was the same.

A meta-analysis showed that RMGIC performed better than conventional GIC for class II restorations in primary teeth^9^. Another study also observed a better performance of RMGICs compared with conventional GICs and composites for class II primary teeth, arguing that RMGICs combine the best properties of both materials: on the one hand they have the good mechanical properties of composites and, on the other, the self-adhesive properties of GICs^73^. Vitremer (3 M ESPE, St. Paul, MN, USA) was the RMGIC used in the largest number of studies included in our meta-analysis. Of the five studies, in four it was used together with Vitremer Primer (3 M ESPE, St. Paul, MN, USA), a light cure adhesive that contains, among other things, 2-hydroxyethyl methacrylate monomer (45–55%) and the copolymer of acrylic and itaconic acids (10–30%). The joint use of the Primer gives it a greater adhesive capacity and reduced sensitivity to the exchange of water with the surrounding environment by rapid photopolymerization^72^.

HVGIC showed, in our study, the worst retention rate (0.671), similar to other studies that found a 65% survival rate in multi-surface ART restorations using HVGIC^74^ or failures in 30% of class II ART restorations during the first month using HVGIC^52^. A study that compared the use of HVGIC using ART versus a conventional cavity technique concluded that, for HVGIC, there is a greater risk with ART cavities than with conventional cavities in primary tooth decay treatments, and success rates in classes II are worse than in class I^75^. This association between the poor results of ART and the poor clinical performance of HVGIC is strongly supported by our results, in which the bivariate correlations between predictors revealed a strong relationship (*r* = 0.66, *p* < 0.001) between the cavity form and the type of material. This may be due to the peculiarities of the ART technique, which uses HVGIC as a restorative material and does not allow complete isolation, meaning saliva contamination of the operative field is very frequent^76^ and the survival of multi-surface restorations could be more dependent on the material, operator and control of the operative field than single-surface restorations^57^. However, in a randomized controlled study 10 year follow-up to evaluate the durability and clinical performance of a HVGIC (processed with a resinous coating) compared with a micro filled composite resin in conventional class I y II cavities, in permanent teeth isolated with cotton rolls and suction devices, no significant differences were found for both restorative materials in terms of marginal adaptation, anatomical form, secondary caries, postoperative sensitivity, surface texture, and retention. The HVGIC could be also considered a good alternative to amalgam^77^.

Although we found a higher retention success rate when the dentist was experienced, we found no influence of the type of isolation. In “*in vitro”* studies, adhesion to enamel and dentin of materials containing composites are very sensitive to salivary contamination, although the results of *“in vivo”* studies are unclear. A systematic review found that, in the longevity of direct dental restorations made with a tooth-coloured material in primary teeth, the use of a rubber dam did not influence the results compared with the use of roll of cotton together with a saliva ejector^78^. A meta-analysis concluded that there are few studies with a very low quality of evidence on the advantages of using the rubber dam compared to cotton rolls together with a saliva ejector on the survival of restorations and neither, the previous application of cavity conditioner nor the final application of coating on the glass ionomer, influenced the success of the clinical variables studied^79^.

Our results showed that, of all the methodological variables used, the type of trial, randomization, reporting bias and the source of funding did not influence the retention, marginal integrity, anatomic form and absence of recurrent caries success-rates, indicating that the type of study did not influence the results of the different materials and supports our decision not to use quality scales, where the type of study is decisive in the final score, to decide the inclusion of the works in our meta-analysis, but include all studies that met the inclusion criteria considering as an independent study each of the materials used or each of the different experimental conditions used with the same material.

Evaluator blinding was important in determining the success of the restoration in the four clinical criteria evaluated in the opposite direction as expected, since when the evaluator knew the material the degree of success measured was lower than when they did not. The influence that we have observed in the triple blind indicates that in the next studies that are carried out, the evaluator must be masked. Meta-regression of each moderator variable showed only the sample size had a significant negative association with the estimated retention success rate indicating that, as the sample size increased, the retention success rate fell. One of the limitations of the studies included in our meta-analysis was the small sample size [median 56, min–max 12–210]: studies were initiated with a small sample size with a high loss rate, which was very high at 24 months. Multiple meta-regressions to determine the predictors that explained the success of proximal restorations showed a similar behaviour for retention, marginal integrity, anatomic form, and recurrent caries success rates. All included the cavity form and assessor blinding as predictors. In addition, the sample size, the type of material and the use of coat were predictors of the index of retention.

A possible bias is the different criteria used in the studies to assess the clinical performance of the restorations. Thirteen used the various modifications of the USPHS criteria, one study used the USPHS criteria plus ART criteria, one study used the FDI criteria and one study used an own system. As there may be differences in the assessment of success depending on the criteria used, and the lack of sensitivity of the ART criteria in detecting improvements in the clinical performance of the materials currently used for dental restorations^80^, we unified the criteria used in the selected articles into four categories: retention, marginal integrity, anatomic form and absence of recurrent caries. This enabled comparison of the studies and elimination of the bias that the use of different evaluation systems could introduce.

After RMGIC, the material that presented the best results was the open-type sandwich method followed by resin-containing materials adhered with the total etching technique. The sandwich method, which uses RMGIC as a cavitary base material and composite as a surface material, seems to have the advantages of both materials. However, only one study was included in this meta-analysis, and therefore further studies are required to determine whether the good results are maintained. Likewise, studies that combine the speed and simplicity of the ART technique with RMGICs, which had the best clinical behaviour in proximal restorations at 24 months, are required.

In conclusion, the null hypothesis of our meta-analysis was disproved, as the index of success of proximal restorations in primary teeth at 24 months was found to depend on the type of coloured material used. The materials with the highest success rates were those that contained resin. Of these, RMGIC performed best. The highest failure rate was for HVGIC and with the cavity made using ART, which were significantly correlated. The shape of the cavity, triple blinding and the experience of the operator had the most influence on the success rates of proximal restorations.

## Supplementary information

Supplementary Dataset .

Supplementary file 1.

Supplementary file 2.

Supplementary file 3.

Supplementary file 4.

## Data Availability

All data generated or analysed during this study are included in this published article (and its Supplementary Information files).

## References

[1] Kassebaum NJ (2017). GBD 2015 Oral Health Collaborators. Global, Regional, and National Prevalence, Incidence, and Disability-Adjusted Life Years for Oral Conditions for 195 Countries, 1990–2015: A Systematic Analysis for the Global Burden of Diseases, Injuries, and Risk Factors. J Dent Res..

[2] Corrêa-Faria P, Paixão-Gonçalves S, Paiva SM, Pordeus IA (2016). Incidence of dental caries in primary dentition and risk factors: a longitudinal study. Braz Oral Res..

[3] Alkhtib A (2016). Prevalence of early childhood caries and enamel defects in four and five-year old Qatari preschool children. BMC Oral Health.

[4] Dermata A, Papageorgiou SN, Fragkou S, Kotsanos N (2018). Comparison of resin modified glass ionomer cement and composite resin in class II primary molar restorations: a 2-year parallel randomized clinical trial. Eur Arch Paediatr Dent..

[5] Kielbassa AM, Glockner G, Wolgin M, Glockner K (2016). Systematic Review on Highly Viscous Glass-Ionomer Cement/Resin Coating Restorations (Part I): Do They Merge Minamata Convention and Minimum Intervention Dentistry?. Quintessence Int..

[6] Yildiz E, Simsek M, Pamir Z (2016). Fracture strength of restorations in proximal cavities of primary molars. Scanning.

[7] Milsom KM, Tickle M, Blinkhorn A (2002). The prescription and relative outcomes of different materials used in general dental practice in the north west region of England to restore the primary dentition. J Dent..

[8] Sidhu SK, Nicholson JW (2016). A Review of Glass-Ionomer Cements for Clinical Dentistry. J Funct Biomater..

[9] Chadwick BL, Evans DJ (2007). Restoration of class II cavities in primary molar teeth with conventional and resin modified glass ionomer cements: a systematic review of the literature. Eur Arch Paediatr Dent..

[10] Krämer N, Frankenberger R (2001). Clinical performance of a condensable metal-reinforced glass ionomer cement in primary molars. Br Dent J..

[11] Alves dos Santos MP, Passos M, Luiz RR, Maia LC (2009). A randomized trial of resin-based restorations in class I and class II beveled preparations in primary molars: 24-month results. J Am Dent Assoc..

[12] Ersin NK (2006). A clinical evaluation of resin-based composite and glass ionomer cement restorations placed in primary teeth using the ART approach: results at 24 months. J Am Dent Assoc..

[13] Gurgan S, Kutuk Z, Ergin E, Oztas S, Cakir F (2015). Four-year randomized clinical trial to evaluate the clinical performance of a glass ionomer restorative system. Oper Dent..

[14] Kielbassa AM, Glockner G, Wolgin M, Glockner K (2017). Systematic Review on Highly Viscous Glass-Ionomer Cement/Resin Coating Restorations (Part II): Do They Merge Minamata Convention and Minimum Intervention Dentistry?. Quintessence Int..

[15] Demarco, F.F., Corrêa, M.B., Cenci, M.S., Moraes, R.R. & Opdam, N.J. Longevity of posterior composite restorations: not only a matter of materials. *Dent Mater*. **28**: 87–101 10.1016/j.dental.2011.09.003 (2012). 10.1016/j.dental.2011.09.003 22192253

[16] Andersson-Wenckert I, Folkesson UH, van Dijken JW (1997). Durability of a polyacid-modified composite resin (compomer) in primary molars. A multicenter study. Acta Odontol Scand..

[17] Turgut MD, Tekçiçek M, Olmez S (2004). Clinical evaluation of a polyacid-modified resin composite under different conditioning methods in primary teeth. Oper Dent..

[18] Kavvadia K, Kakaboura A, Vanderas AP, Papagiannoulis L (2004). Clinical evaluation of a compomer and an amalgam primary teeth class II restorations: a 2-year comparative study. Pediatr Dent..

[19] Soncini JA, Maserejian NN, Trachtenberg F, Tavares M, Hayes C (2007). The longevity of amalgam versus compomer/composite restorations in posterior primary and permanent teeth: findings From the New England Children’s Amalgam Trial. J Am Dent Assoc..

[20] Sengul F, Gurbuz T (2015). Clinical Evaluation of Restorative Materials in Primary Teeth Class II Lesions. J Clin Pediatr Dent..

[21] Chisini LA (2018). Restorations in primary teeth: a systematic review on survival and reasons for failures. Int J Paediatr Dent..

[22] Toh SL, Messer LB (2007). Evidence-based Assessment of Tooth-Colored Restorations in Proximal Lesions of Primary Molars. Pediatr Dent..

[23] Yengopal V, Harneker SY, Patel N, Siegfried N (2009). Dental fillings for the treatment of caries in the primary dentition. Cochrane Database Syst Rev..

[24] Santos AP (2016). Survival of Adhesive Restorations for Primary Molars: A Systematic Review and Metaanalysis of Clinical Trials. Pediatr Dent..

[25] Moher D, Liberati A, Tetzlaff J, Altman DG (2010). The PRISMA group (2010) Preferred reporting items for systematic reviews and meta-analyses: the PRISMA statement. Int J Surg..

[26] Egger, M., Davery-Smith, G. & Altman, D. Systematic reviews in Health care: Metaanalysis in Context. 2nd ed. London, United Kingdom: BMJ Books (2001).

[27] Atieh M (2008). Stainless steel crown versus modified open-sandwich restorations for primary molars: a 2-year randomized clinical trial. Int J Paediatr Dent..

[28] Andersson-Wenckert I, van Dijken JW, Stenberg R (1995). Effect of cavity form on the durability of glass ionomer cement restorations in primary teeth: a three-year clinical evaluation. ASDC J Dent Child.

[29] Espelid I, Tveit AB, Tornes KH, Alvheim H (1999). Clinical behaviour of glass ionomer restorations in primary teeth. J Dent..

[30] Attin T, Opatowski A, Meyer C, Zingg-Meyer B, Mönting JS (2000). Class II restorations with a polyacid-modified composite resin in primary molars placed in a dental practice: results of a two-year clinical evaluation. Oper Dent..

[31] Ersin NK (2008). The effect of a chlorhexidine containing cavity disinfectant on the clinical performance of high-viscosity glass-ionomer cement following ART: 24-month results. Am J Dent..

[32] Topaloglu-Ak A, Eden E, Frencken JE, Oncag O (2009). Two years survival rate of class II composite resin restorations prepared by ART with and without a chemomechanical caries removal gel in primary molars. Clin Oral Investig..

[33] Folkesson UH, Andersson-Wenckert IE, Van Dijken JWV (1999). Resin-modified glass ionomer cement restorations in primary molars. Swed Dent J..

[34] Andersson-Wenckert I, Sunnegårdh-Grönberg K (2006). Flowable resin composite as a class II restorative in primary molars: A two-year clinical evaluation. Odontol Scand Act..

[35] Kotsanos N, Arizos S (2011). Evaluation of a resin modified glass ionomer serving both as indirect pulp therapy and as restorative material for primary molars. Eur Arch Paediatr Dent..

[36] Lo ECM, Luo Y, Fan MW, Wei SH (2001). Clinical investigation of two glass-ionomer restoratives used with the atraumatic restorative treatment approach in China: two-years results. Caries Res..

[37] Carvalho TS, Sampaio FC, Diniz A, Bönecker M, Van Amerongen WE (2010). Two years survival rate of Class II ART restorations in primary molars using two ways to avoid saliva contamination. Int J Paediatr Dent..

[38] Borenstein M, Hedges LV, Higgins JP, Rothstein HR (2010). A basic introduction to fixed-effect and random-effects models for meta-analysis. Res Synth Methods..

[39] DerSimonian R, Laird N (1986). Meta-analysis in clinical trials. Control Clin Trials..

[40] Wilson, D.B. & Lipsey, M.W. Practical meta-analysis Thousand Oaks, CA: Sage Publications (2001).

[41] Sánchez Meca J, Marín Martínez F (2008). Confidence intervals for the overall effect size in random-effects meta-analysis. Psychol Methods..

[42] Huedo Medina TB, Sánchez Meca J, Marín Martínez F (2006). & Bottle, J. Assessing heterogeneity in meta-analysis: Q statistic or I² index?. Psychol Methods..

[43] Duval S, Tweedie R (2000). Trim and fill: a simple funnel–plot–based method of testing and adjusting for publication bias in meta–analysis. Biometrics..

[44] Rubio Aparicio M, Sánchez Meca J, López López JA, Bottle J, Marín Martínez F (2017). Analysis of categorical moderators in mixed - effects meta - analysis: Consequences of using pooled versus separate estimates of the residual between - studies variances. Br J Math Stat Psychol..

[45] Viechtbauer W, López López JA, Sánchez Meca J, Marín Martínez F (2015). A comparison of procedures to test for moderators in mixed-effects meta-regression models. Psychol Methods..

[46] Knapp G, Hartung J (2003). Improved tests for a random effects meta - regression with a single covariate. Stat Med..

[47] López López JA, Marín Martínez F, Sánchez Meca J, Van den Noortgate W, Viechtbauer W (2014). Estimation of the predictive power of the model in mixed - effects meta - regression: A simulation study. Br J Math Stat Psychol..

[48] Viechtbauer W (2010). Conducting meta-analyses in R with the metafor package. J Stat Softw..

[49] Ermis RB, Kam O, Celik EU, Temel UB (2009). Clinical evaluation of a two-step etch&rinse and a two-step self-etch adhesive system in Class II restorations: two-year results. Oper Dent.

[50] Baracco B, Perdigão J, Cabrera E, Giráldez I, Ceballos L (2012). Clinical evaluation of a low-shrinkage composite in posterior restorations: one-year results. Oper Dent.

[51] Bernardo M (2007). Survival and reasons for failure of amalgam versus composite posterior restorations placed in a randomized clinical trial. J Am Dent Assoc.

[52] Da Franca C, Colares V, Van Amerongen E (2011). Two-year evaluation of the atraumatic restorative treatment approach in primary molars class I and II restorations. Int J Paediatr Dent..

[53] Alves dos Santos MP, Luiz RR, Maia LC (2010). Randomized trial of resin-based restorations in Class I and Class II beveled preparations in primary molars: 48-month results. J Dent..

[54] Webman M (2016). A retrospective study of the 3-year survival rate of resin-modified glass-ionomer cement class II restorations in primary molars. J Clin Pediatr Dent.

[55] Eden E, Topaloglu-Ak A, Frencken JE (2006). Hof van’t M. Survival of self-etch adhesive Class II composite restorations using ART and conventional cavity preparations in primary molars. Am J Dent.

[56] Bücher K, Metz I, Pitchika V, Hickel R, Kühnisch J (2015). Survival characteristics of composite restorations in primary teeth. Clin Oral Investig.

[57] Yu C, Gao XJ, Deng DM, Yip HK, Smales RJ (2004). Survival of glass ionomer restorations placed in primary molars using atraumatic restorative treatment (ART) and conventional cavity preparations: 2-year results. Int Dent J..

[58] Pinto GDS (2014). Longevity of posterior restorations in primary teeth: results from a paediatric dental clinic. J Dent.

[59] Dalpian DM (2014). Clinical and radiographic outcomes of partial caries removal restorations performed in primary teeth. Am J Dent.

[60] Kupietzky A, Joachim DA, Tal E, Moskovitz M (2019). Long-term clinical performance of heat-cured high-viscosity glass ionomer class II restorations versus resin-based composites in primary molars: a randomized comparison trial. Eur Arch Paediatr Dent.

[61] Alyahya A, Khanum A, Qudeimat M (2018). Clinical assessment of class II resin-based composites versus preformed metal crowns performed on primary molars in patients at high risk of caries. Eur Arch Paediatr Dent.

[62] Pummer A (2020). Longevity of posterior composite and compomer restorations in children placed under different types of anesthesia: a retrospective 5-year study. Clin Oral Invest.

[63] Guzmán ARD (1995). Comportamiento clínico de un vidrio ionomérico tipo Cermet en odontopediatría. Acta odontol venez..

[64] Ji M, Lee S, Lee N (2015). Retrospective Study of Survival Rates According to the Type of Dental Restoration of Proximal Caries in Primary Molars. J Korean. Acad Pediatr Dent.

[65] Varpio M, Warfvinge J, Norén JG (1990). Proximo-occlusal composite restorations in primary molars: marginal adaptation, bacterial penetration, and pulpal reactions. Acta Odontol Scand.

[66] Attin T (2001). Three-year follow up assessment of Class II restorations in primary molars with a polyacid-modified composite resin and a hybrid composite. Am J Dent.

[67] US Department of Health and Human Services. Final RuleFederal Register 75: Issue 112 (Friday, June 11, 2010). Available at: “http://www. fda. gov/downloads/medicaldevices/productsandmedicalprocedures/dentalproducts/dentalamalgam/ucm174024. pdf”. Accesed June 25, 2019.

[68] Regulation (EU) 2017/852 of the European Parliament and of the Council of 17 May 2017 on mercury, and repealing Regulation (EC) No 1102/2008. Available at: “https://eur-lex. europa. eu/legal-content/EN/TXT/PDF/?uri=CELEX:32017R0852&from=EN”. Accesed June 25, 2019.

[69] van ‘t Hof, M. A., Frencken, J. E., van Palenstein Helderman, W. H. & Holmgren, C. J. The atraumatic restorative treatment (ART) approach for managing dental caries: a meta-analysis. *Int Dent J*. **56**:345–51, 10.1111/j.1875-595x.2006.tb00339.x (2006). 10.1111/j.1875-595x.2006.tb00339.x 17243467

[70] Bonifácio CC (2013). Survival Rate of approximal-ART Restorations Using a Two-Layer Technique for Glass Ionomer Insertion. Clin Oral Investig..

[71] Hesse D (2016). Bilayer technique and nano-filled coating increase success of approximal ART restorations: a randomized clinical trial. Int J Paediatr Dent..

[72] Qvist V, Laurberg L, Poulsen A, Teglers PT (2004). Class II restorations in primary teeth: 7-year study on three resin-modified glass ionomer cements and a compomer. Eur J Oral Sci..

[73] Dias AGA (2018). Clinical performance of glass ionomer cement and composite resin in Class II restorations in primary teeth: A systematic review and meta-analysis. J Dent..

[74] De Amorim RG (2018). Survival percentages of atraumatic restorative treatment (ART) restorations and sealants in posterior teeth: an updated systematic review and meta-analysis. Clin Oral Investig..

[75] Dorri M (2017). Atraumatic restorative treatment versus conventional restorative treatment for managing dental caries. Cochrane Database Syst Rev..

[76] Frencken, J.E., Pilot, T., Songpaisan, Y. & Phantumvanit, P. Atraumatic restorative treatment (ART): rationale, technique, and development. *J Public Health Dent*. **56** (3 Spec No):135–40; discussion 161–3 (1996). 10.1111/j.1752-7325.1996.tb02423.x 8915958

[77] Gurgan S, Kutuk ZB, Cakir FY, Ergin E (2020). A Randomized Controlled 10 Years Follow Up of a Glass Ionomer Restorative Material in Class I and Class II Cavities. J Dent..

[78] Rodrigues Cajazeira M, De Sabóia TM, Maia LC (2014). Influence of the operatory field isolation technique on tooth-colored direct dental restorations. Am J Dent..

[79] Wang, Y. Rubber dam isolation for restorative treatment in dental patients. *Cochrane Database Syst Rev*. **20**; 9: CD009858 10.1002/14651858.CD009858.pub2 (2016). 10.1002/14651858.CD009858.pub2 PMC6457832 27648846

[80] De Amorim RG, Leal SC, Frencken JE (2012). Survival of atraumatic restorative treatment (ART) sealants and restorations: a meta-analysis. Clin Oral Investig..

