# Supplementary material for: Success rate of proximal tooth-coloured direct restorations in primary teeth at 24 months: a meta-analysis
Source: Sci Rep. 2020 Apr 14;10:6409. doi: 10.1038/s41598-020-63497-4 (PMC7156457; doi:10.1038/s41598-020-63497-4)
Supplement: Supplementary file 3 — Supplementary file 2. [file 41598_2020_63497_MOESM3_ESM.pdf]

"Success rate of proximal tooth-coloured direct restorations in primary teeth at 24 months: a meta-analysis"

Antonio J. Ortiz-Ruiz, Nuria Pérez-Guzmán, María Rubio-Aparicio , Julio Sánchez-Meca

Supplementary file S2

Supplementary Table 1. Evaluation criteria of the different systems published and their equivalents with the four categories used in our study.

		**Criteria of our study**			
		**Retention**	**Marginal integrity**	**Anatomic form**	**Absence of recurrent caries**
USPHS criteria by Ryge and Cvar [10, 25], USPHS modified by Ryge and Snyder [26], USPHS modified by Ryge [11, 16, 27, 28, 29, 30]	Marginal discoloration		X		
	Marginal adaptation		X		
	Marginal caries				X
	Anatomic form			X	
	Surface texture			X	
	Proximal contact				
	Axial contour			X	
	Gingival health				
	Occlusion			X	
	Postoperative hypersensitivity				
	Retention	X			
	Bulk fracture	X			
	Color matching				
	Sensitivity				
USPHS criteria modified by Van Dijken [14, 31, 32, 33]	Retention	X			
	Anatomic form			X	
	Marginal adaptation		X		
	Recurrent caries				X
ART modified criteria [34]	Present and good (code 0)				
	Present, marginal defect or wear 0.5 mm (code 1)		X		
	Wear of surface 0.5 mm (code 2)			X	
	Defect at margin 0.5 mm (code 3)		X		
	Combination of above (code 4)			X	
	Almost) not present (code 5)	X			
	Other treatment performed (code 6)	X			
Own system	Successful treatment: it was still present		X		

[35]:	and correct or having only a slight wear or defect at the margin less than 0.5 mm in depth.				
	Treatment failures: the restorations were completely lost or fractured with defects 0.5 mm in depth or greater. Secondary caries or inflammation of the pulp.	X			
	Lost to followup				
FDI criteria [18].	Surface luster				
	Surface staining				
	Colour stability and translucency				
	Anatomic form			X	
	Fractures and retention	X			
	Marginal adaptation		X		
	Contact point (food impact)				
	Radiographic examination				
	Patient´s view				
	(Hyper-) sensitivity, tooth vitality				
	Recurrence of caries				X
	Tooth integrity				
	Periodontal response				
	Adjacent mucosa				
	Oral and general health				
