# Supplementary material for: Success rate of proximal tooth-coloured direct restorations in primary teeth at 24 months: a meta-analysis
Source: Sci Rep. 2020 Apr 14;10:6409. doi: 10.1038/s41598-020-63497-4 (PMC7156457; doi:10.1038/s41598-020-63497-4)
Supplement: Supplementary file 4 — Supplementary file 3. [file 41598_2020_63497_MOESM4_ESM.pdf]

"Success rate of proximal tooth-coloured direct restorations in primary teeth at 24 months: a meta-analysis"

Antonio J. Ortiz-Ruiz, Nuria Pérez-Guzmán, María Rubio-Aparicio , Julio Sánchez-Meca

Supplementary file S3

Supplementary figures

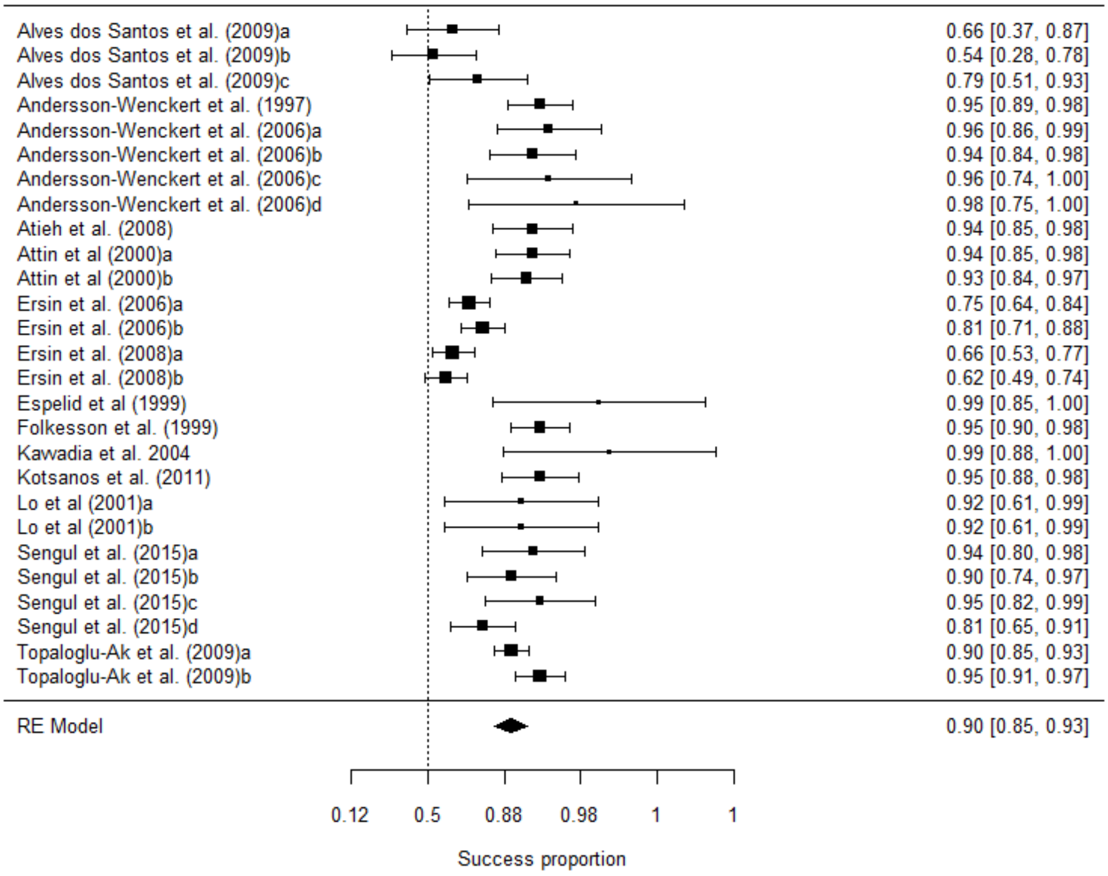

Supplementary figure 1. Forest plot displaying the success rates at 24 months for marginal integrity (and 95% confidence intervals).

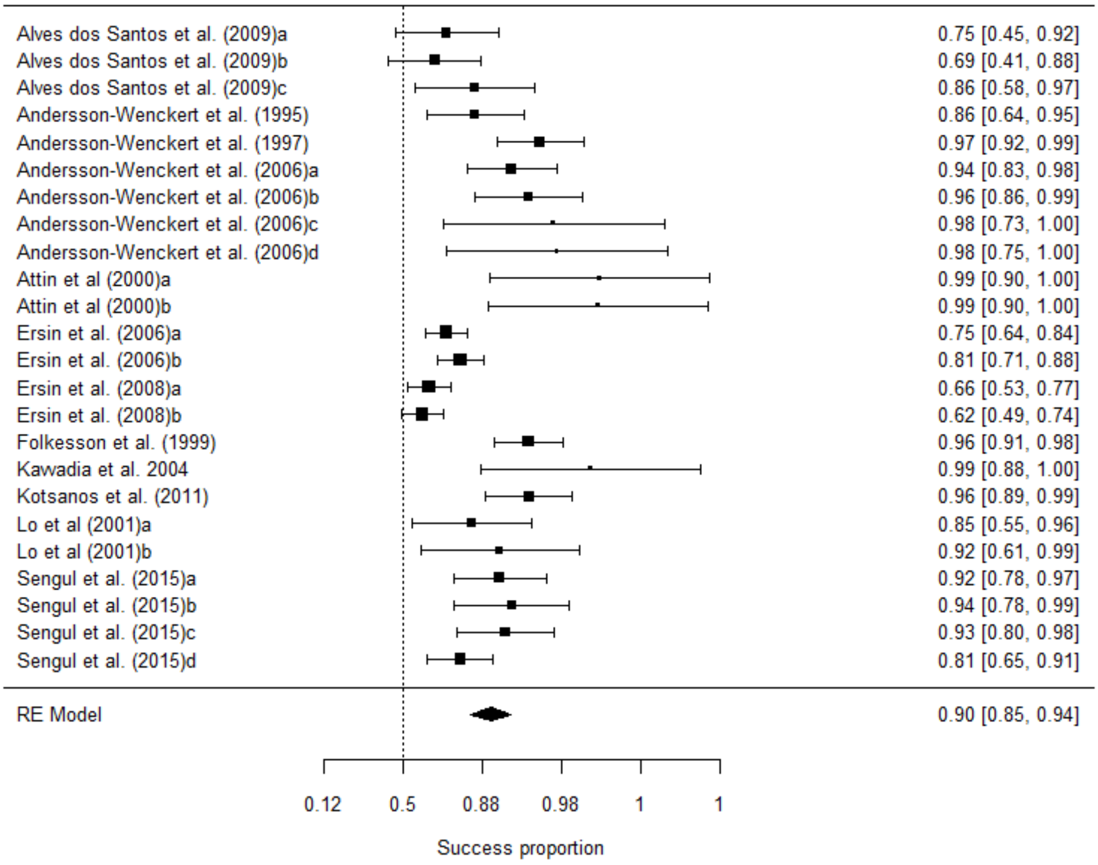

Supplementary figure 2. Forest plot displaying the success rates at 24 months for anatomic form (and 95% confidence intervals).

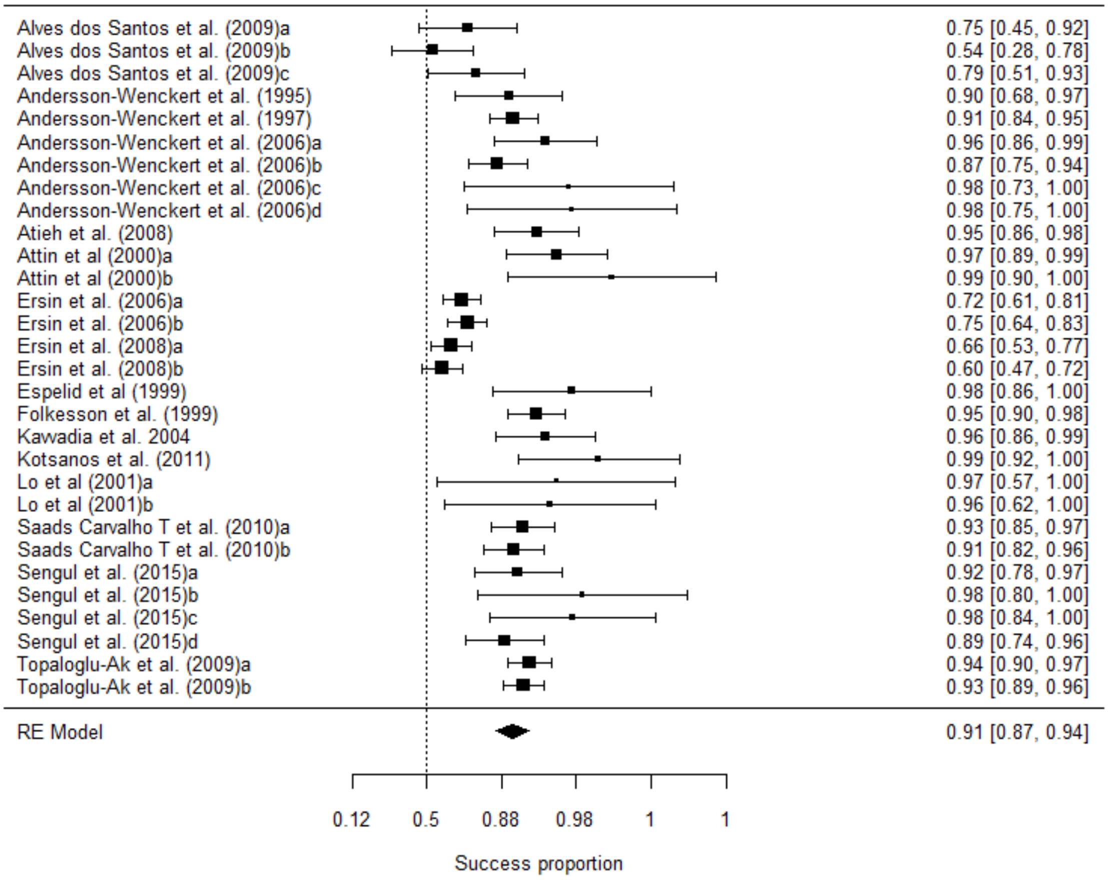

Supplementary figure 3. Forest plot displaying the success rates at 24 months for recurrent caries (and 95% confidence intervals).

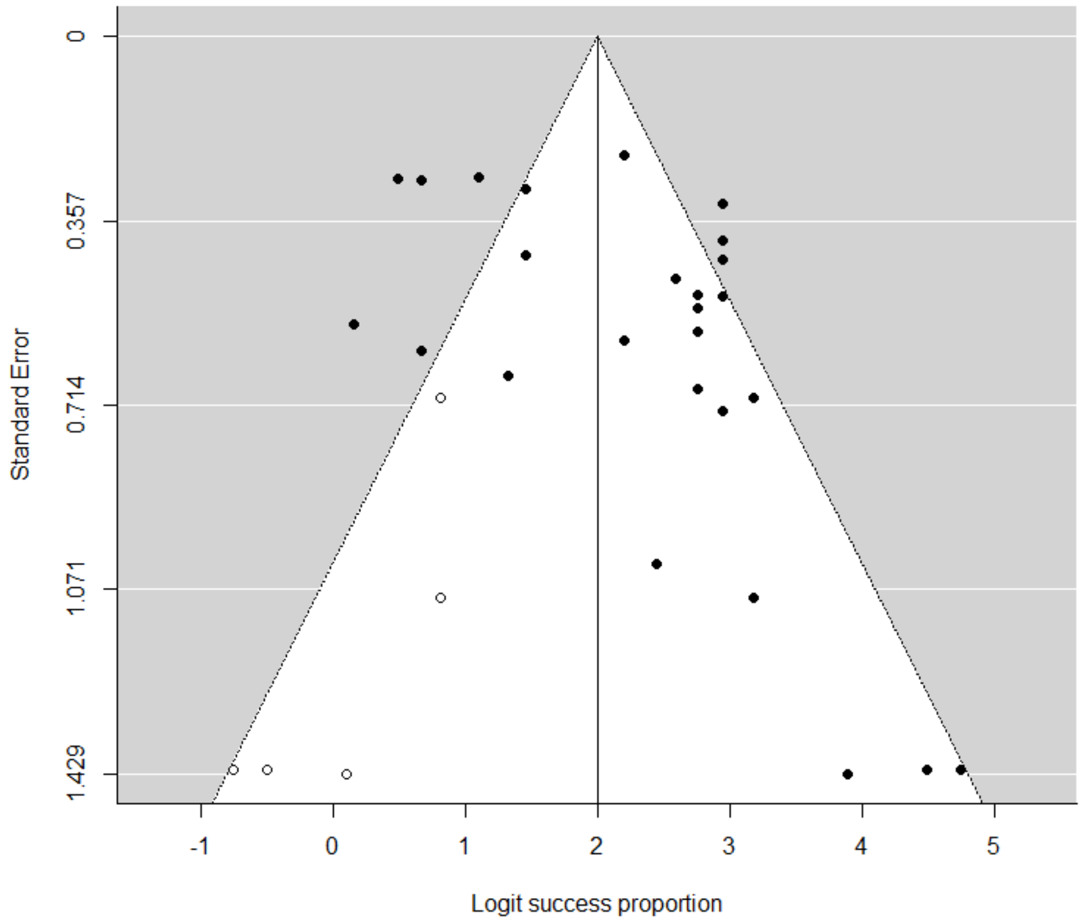

Supplementary figure 4. Funnel plot of the marginal integrity prevalence logits at 24 months. The five white circles are imputed logits by means of the Duval and Tweedie's trim-and-fill method.

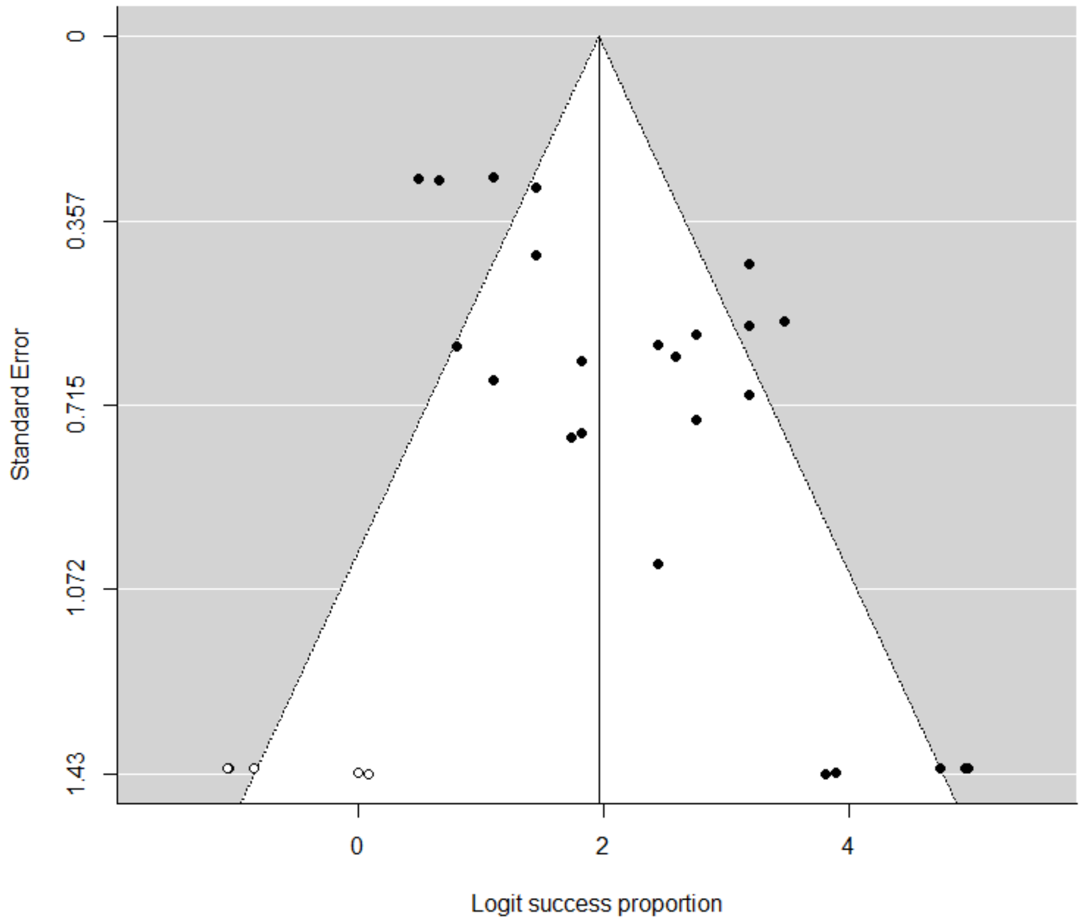

Supplementary figure 5. Funnel plot of the anatomic form prevalence logits at 24 months. The five white circles are imputed logits by means of the Duval and Tweedie's trim-and-fill method.

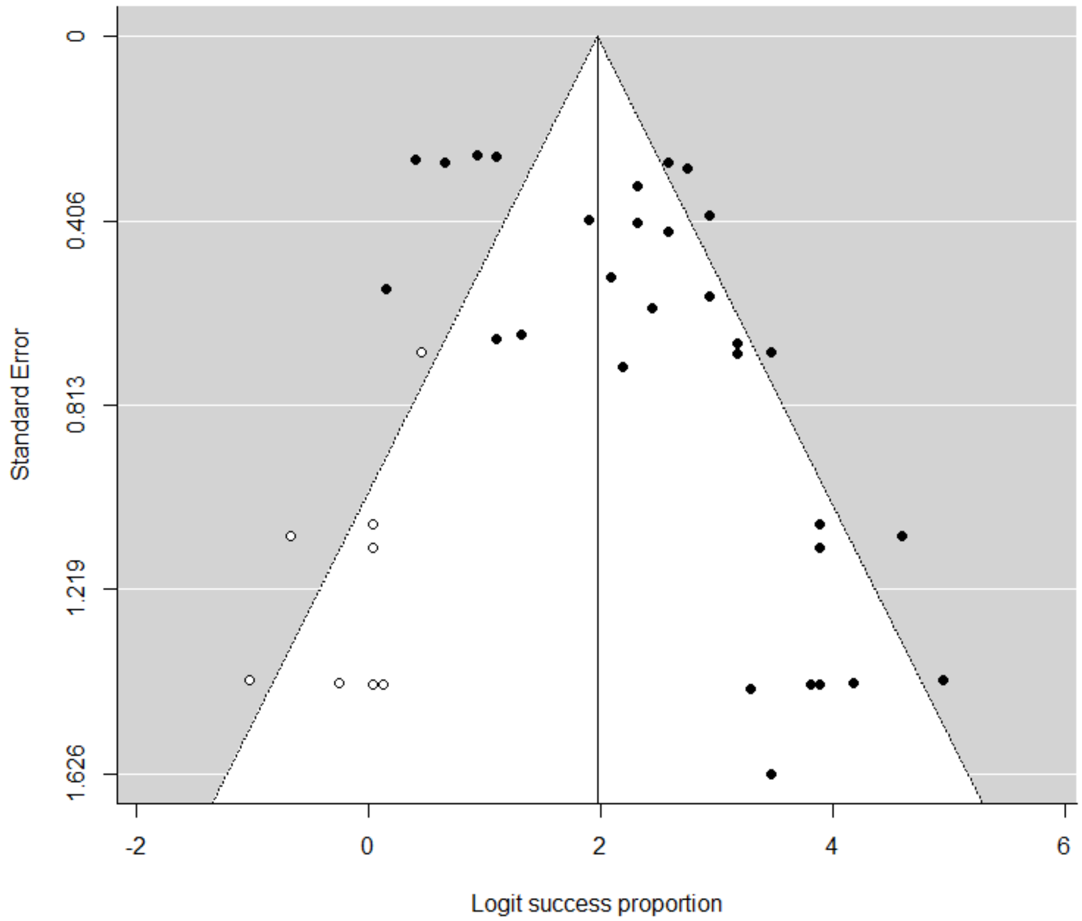

Supplementary figure 6. Funnel plot of the recurrent caries prevalence logits at 24 months. The eight white circles are imputed logits by means of the Duval and Tweedie's trim-and-fill method.
