# Supplementary material for: Success rate of proximal tooth-coloured direct restorations in primary teeth at 24 months: a meta-analysis
Source: Sci Rep. 2020 Apr 14;10:6409. doi: 10.1038/s41598-020-63497-4 (PMC7156457; doi:10.1038/s41598-020-63497-4)
Supplement: Supplementary file 5 — Supplementary file 4. [file 41598_2020_63497_MOESM5_ESM.pdf]

“Success rate of proximal tooth-coloured direct restorations in primary teeth at 24 months: a meta-analysis”

Antonio J. Ortiz-Ruiz, Nuria Pérez-Guzmán, María Rubio-Aparicio , Julio Sánchez-Meca

Supplementary file S4

Supplementary Tables

Supplementary Table 1. Results of the simple meta-regressions of continuous moderator variables on the marginal integrity prevalence estimated

Moderator variable	k	b_j	F	p	Q_E	R^2
Mean age (years)	23	0.106	0.196	.663	115.088***	0.0
SD of age (years)	12	-0.477	0.532	.483	30.270***	.13
Sample size	27	-0.001	0.348	.560	129.693***	0.0
Gender (% male)	16	-0.039	1.045	.324	75.891***	.03
Year of the study	27	-0.061	2.338	.139	120.447***	.04

k = number of studies. b_j = regression coefficient of each predictor. F = Knapp-Hartung’s statistic for testing the significance of the predictor (the degrees of freedom for this statistic are 1 for the numerator and $k - 2$ for the denominator). p = probability level for the F statistic. Q_E = statistic for testing the model misspecification. R^2 = proportion of variance accounted for by the predictor. *** $p < .0001$.

Supplementary Table 2. Results of the weighted ANOVAs of qualitative moderator variables on the marginal integrity prevalence estimated

Moderator variable	k	p_+	95% CI LL	95% CI LU	ANOVA results
-Material:					$F(4,22) = 2.22, p = .100$
Resin-based material + total-etching adhesion	7	.898	.796	.952	$R^2 = .49$
					$Q_W(22)=64.880, p<.001$
Resin-based material + self-etching adhesion	8	.916	.848	.956	
Resin-modified Glass-ionomer cement (RMGIC)	6	.927	.843	.968	
High-viscosity Glass-ionomer Cement (HVGIC)	5	.743	.553	.871	
Open Sandwich Restoration	1	.940	.695	.991	
-Use of coat:					$F(1,10) = 2.32, p = .158$
No	7	.916	.803	.966	$R^2 = .44$
Yes	5	.804	.595	.919	$Q_W(10)=37.68, p<.001$
-Use of cavity conditioner:					$F(1,10) = 2.32, p = .159$
No	2	.963	.779	.995	$R^2 = .10$
Yes	10	.857	.737	.928	$Q_W(10)=56.58, p<.001$
-Use of rubber dam isolation:					$F(1,22) = 2.25, p = .148$
No	14	.899	.833	.941	$R^2 = .16$
Yes	10	.883	.782	.941	$Q_W(22)=206.44, p<.001$
-Cavity form:					$F(1,25) = 4.67, p = .040$

Atraumatic restorative treatment (ART)	8	.831	.716	.905	R^2 = .21 $Q_W(25)$=102.11, p<.001
Conventional cavity design	19	.921	.877	.950	
-Type of study:					
Noncontrolled trial	3	.950	.863	.983	$F(2,24)$ = 2.03, p = .153
Nonrandomized controlled trial	11	.855	.763	.914	R^2 = .34
Randomized controlled trial	13	.907	.838	.948	$Q_W(24)$=83.76, p<.001
-Random assignment:					$F(1,25)$ = 0.28, p = .603
No/Incorrect	14	.888	.819	.933	R^2 = .04
Correct	13	.909	.837	.951	$Q_W(25)$=116.83, p<.001
-Triple-blind					$F(1,25)$ = 5.82, p = .023
No	11	.938	.891	.966	R^2 = .21
Yes	16	.857	.785	.908	$Q_W(25)$=102.64, p<.001
-Reporting bias:					$F(1,25)$ = 0.52, p = .477
Yes	2	.935	.767	.984	R^2 = .01
No	25	.893	.843	.929	$Q_W(25)$=123.80, p<.001
-Financial source:					$F(1,12)$ = 0.14, p = .717
Private or mixed	5	.875	.671	.959	R^2 = .07
Public	9	.900	.788	.956	$Q_W(12)$=41.97, p<.001

k = number of studies. p_+ = mean prevalence. LL and LU = lower and upper 95% confidence limits for p_+. F = Knapp-Hartung's statistic for testing the significance of the moderator variable. Q_W = statistic for testing the model misspecification. R^2 = proportion of variance accounted for by the moderator.

Supplementary Table 3. Results of the simple meta-regressions of continuous moderator variables on the anatomic form prevalence estimated

Moderator variable	k	b_j	F	p	Q_E	R^2
Mean age (years)	20	0.099	0.154	.699	90.226***	0.0
SD of age (years)	8	-0.560	0.227	.651	20.433***	0.0
Sample size	24	-0.008	4.239	.052	82.688***	.17
Gender (% male)	13	0.000	0.000	.996	52.569***	.24
Year of the study	24	-0.058	1.830	.189	95.783***	.05

k = number of studies. b_j = regression coefficient of each predictor. F = Knapp-Hartung's statistic for testing the significance of the predictor (the degrees of freedom for this statistic are 1 for the numerator and $k - 2$ for the denominator). p = probability level for the F statistic. Q_E = statistic for testing the model misspecification. R^2 = proportion of variance accounted for by the predictor. *** $p < .0001$.

Supplementary Table 4. Results of the weighted ANOVAs of qualitative moderator variables on the anatomic form prevalence estimated

Moderator variable	k	p_+	95% CI LL	95% CI LU	ANOVA results
-Material:					
Resin-based material + total-etching adhesion	7	.919	.821	.966	$F(3,20) = 3.08, p = .051$ $R^2 = .51$
Resin-based material + self-etching adhesion	6	.926	.829	.970	$Q_W(20)=51.936, p<.001$
Resin-modified Glass-ionomer cement (RMGIC)	5	.934	.848	.973	
High-viscosity Glass-ionomer Cement (HVGIC)	6	.759	.586	.875	
-Use of coat:					$F(1,9) = 3.72, p = .086$
No	6	.916	.808	.966	$R^2 = .49$
Yes	5	.785	.608	.896	$Q_W(9)=29.87, p<.001$
-Use of cavity conditioner:					$F(1,20) = 0.06, p = .816$
No	13	.908	.824	.954	$R^2 = 0.0$
Yes	9	.896	.779	.955	$Q_W(20)=98.452, p<.001$
-Cavity form:					$F(1,22) = 13.75, p = .001$
Atraumatic restorative treatment (ART)	6	.749	.606	.853	$R^2 = .63$ $Q_W(22)=49.23, p<.001$
Conventional cavity design	18	.929	.889	.956	
-Type of study:					
Noncontrolled trial	4	.950	.867	.982	$F(2,21) = 2.22, p = .133$

Nonrandomized controlled trial	10	.844	.731	.916	R^2 = .45
Randomized controlled trial	10	.907	.806	.958	$Q_W(21)$=61.331, p<.001
-Random assignment:					$F(1,22)$ = 0.13, p = .724
No/Incorrect	14	.895	.817	.942	R^2 = .01
Correct	10	.912	.809	.962	$Q_W(22)$=98.93, p<.001
-Triple-blind					$F(1,22)$ = 5.23, p = .032
No	10	.943	.886	.972	R^2 = .33
Yes	14	.852	.760	.912	$Q_W(22)$=75.55, p<.001
	21	.892	.829	.932	
-Reporting bias:					$F(1,22)$ = 1.88, p = .184
No	21	.892	.829	.932	R^2 = .02
Yes	3	.964	.828	.994	$Q_W(22)$=99.64, p<.001
-Financial source:					$F(1,13)$ = 1.51, p = .241
Private or mixed	5	.850	.678	.939	R^2 = .34
Public	10	.919	.846	.959	$Q_W(13)$=30.86, p=.004

k = number of studies. p_+ = mean prevalence. LL and LU = lower and upper 95% confidence limits for p_+. *F* = Knapp-Hartung's statistic for testing the significance of the moderator variable. Q_W = statistic for testing the model misspecification. R^2 = proportion of variance accounted for by the moderator.

Supplementary Table 5. Results of the simple meta-regressions of continuous moderator variables on the recurrent caries prevalence estimated

Moderator variable	k	b_j	F	p	Q_E	R^2
Mean age (years)	26	-0.152	0.443	.512	114.693***	.15
SD of age (years)	12	-0.783	1.024	.336	30.253***	.43
Sample size	30	-0.002	0.715	.405	151.272***	0.0
Gender (% male)	18	-0.002	0.002	.964	76.943***	.19
Year of the study	30	-0.035	0.671	.419	148.87***	0.0

k = number of studies. b_j = regression coefficient of each predictor. F = Knapp-Hartung's statistic for testing the significance of the predictor (the degrees of freedom for this statistic are 1 for the numerator and $k - 2$ for the denominator). p = probability level for the F statistic. Q_E = statistic for testing the model misspecification. R^2 = proportion of variance accounted for by the predictor. *** $p < .0001$.

Supplementary Table 6. Results of the weighted ANOVAs of qualitative moderator variables on the recurrent caries prevalence estimated

Moderator variable	k	p_+	95% CI LL	95% CI LU	ANOVA results
-Material:					$F(4,25) = 1.56, p = .216$
Resin-based material + total-etching adhesion	7	.894	.771	.954	$R^2 = .26$<br>$Q_W(25)=99.41, p<.001$
Resin-based material + self-etching adhesion	8	.924	.847	.963	
Resin-modified Glass-ionomer cement (RMGIC)	6	.954	.879	.983	
High-viscosity Glass-ionomer Cement (HVGIC)	8	.831	.692	.915	
Open Sandwich Restoration	1	.950	.677	.994	
-Use of coat:					$F(1,13) = 4.97, p = .044$
No	9	.935	.862	.971	$R^2 = .65$
Yes	6	.801	.619	.909	$Q_W(13)=35.21, p<.001$
-Use of cavity conditioner:					$F(1,13) = 1.46, p = .248$
No	2	.965	.787	.995	$R^2 = .09$
Yes	13	.892	.796	.946	$Q_W(13)=74.86, p<.001$
-Use of rubber dam isolation:					$F(1,25) = 0.09, p = .762$
No	16	.899	.829	.942	$R^2 = 0.0$
Yes	11	.911	.825	.957	$Q_W(25)=142.60, p<.001$
-Cavity form:					$F(1,28) = 4.37, p = .046$
Atraumatic restorative treatment (ART)	10	.853	.751	.918	$R^2 = .16$<br>$Q_W(28)=125.87, p<.001$
Conventional cavity design	20	.932	.889	.959	

-Dentist experience:					$F(1,28) = 0,04$, $p = .844$
Experienced	28	.908	.861	.941	$R^2 = 0.0$
Non-experienced	2	.921	.711	.982	$Q_W(28)=147.48$, $p<.001$
-Type of study:					
Noncontrolled trial	4	.942	.837	.981	$F(2,24) = 2.03$, $p = .153$
Nonrandomized controlled trial	11	.865	.759	.929	$R^2 = .34$
Randomized controlled trial	15	.913	.849	.951	$Q_W(24)=83.76$, $p<.001$
-Random assignment:					$F(1,28) = 0.16$, $p = .695$
No/Incorrect	15	.899	.828	.943	$R^2 = .17$
Correct	15	.914	.849	.953	$Q_W(28)=123.21$, $p<.001$
-Triple-blind					$F(1,28) = 11.92$, $p = .002$
No	12	.958	.924	.978	$R^2 = .28$
Yes	18	.859	.794	.906	$Q_W(28)=113.74$, $p<.001$
-Reporting bias:					$F(1,28) = 1.76$, $p = .196$
No	27	.902	.853	.935	$R^2 = .04$
Yes	3	.963	.847	.992	$Q_W(28)=144.68$, $p<.001$
-Financial source:					$F(1,15) = 0.55$, $p = .471$
Private or mixed	5	.851	.679	.939	$R^2 = .27$
Public	12	.895	.822	.941	$Q_W(15)=41.32$, $p<.001$

k = number of studies. p_+ = mean prevalence. LL and LU = lower and upper 95% confidence limits for p_+. *F* = Knapp-Hartung's statistic for testing the significance of the moderator variable. Q_W = statistic for testing the model misspecification. R^2 = proportion of variance accounted for by the moderator.

Supplementary Table 7. Results of the multiple meta-regression model applied on the marginal integrity success rates, taking as predictors the cavity form, and the assessor blinding (k = 27).

Predictor variable	b_j	t	p	Model fit
Intercept	2.442	1.73	.097	$F(2, 24) = 3.89, p = .051$
Cavity form	0.471	0.97	.343	$R^2 = .22$
Assessor blinding	-0.661	-1.39	.178	$Q_E(24) = 97.868, p < .0001$

b_j = regression coefficient of each predictor. t = statistic for testing the significance of the predictor (with 24 degrees of freedom). p = probability level for the t statistic. F = Knapp-Hartung's statistic for testing the significance of the full model. Q_E = statistic for testing the model misspecification.

Supplementary Table 8. Results of the multiple meta-regression model applied on the anatomic form success rates, taking as predictors the cavity form, and the assessor blinding (k = 24).

Predictor variable	b_j	t	p	Model fit
Intercept	0.538	0.38	.711	$F(2, 21) = 6.908, p = .005$
Cavity form	1.277	2.59	.017	$R^2 = .61$
Assessor blinding	-0.358	-0.74	.469	$Q_E(21) = 48.701, p < .0001$

b_j = regression coefficient of each predictor. t = statistic for testing the significance of the predictor (with 21 degrees of freedom). p = probability level for the t statistic. F = Knapp-Hartung's statistic for testing the significance of the full model. Q_E = statistic for testing the model misspecification.

Supplementary Table 9. Results of the multiple meta-regression model applied on the recurrent caries success rates, taking as predictors the cavity form, and the assessor blinding (k = 15).

Predictor variable	b_j	t	p	Model fit
Intercept	6.33	2.47	.031	$F(3, 11) = 6.976$, $p = .007$
Use of coat	-1.24	-2.66	.022	$R^2 = .83$
Cavity form	-0.041	-0.06	.957	$Q_E(11) = 20.244$, $p = .042$
Assessor blinding	-1.453	-1.86	.086	

b_j = regression coefficient of each predictor. t = statistic for testing the significance of the predictor (with 11 degrees of freedom). p = probability level for the t statistic. F = Knapp-Hartung's statistic for testing the significance of the full model. Q_E = statistic for testing the model misspecification.
